# Universal Newborn Hearing Screening Program: 10-Year Outcome and Follow-Up from a Screening Center in Germany

**DOI:** 10.3390/ijns9040061

**Published:** 2023-10-23

**Authors:** Kruthika Thangavelu, Kyriakos Martakis, Silke Feldmann, Bernhard Roth, Peter Herkenrath, Ruth Lang-Roth

**Affiliations:** 1 Department of Otorhinolaryngology, Head and Neck Surgery, University Hospital Marburg, Philipps-University Marburg, Baldingerstrasse, 35043 Marburg, Germany; 2 Department of Pediatric Neurology, Social Pediatrics and Epileptology, Justus-Liebig-University Giessen and University Hospital Marburg Giessen, Feulgenstrasse 10–12, 35392 Giessen, Germany; kyriakos.martakis@paediat.med.uni-giessen.de; 3 Department of Pediatrics, Faculty of Medicine and University Hospital Cologne, Kerpener Strasse 62, 50937 Cologne, Germany; peter.herkenrath@uk-koeln.de; 4 Department of Otorhinolaryngology, Head and Neck Surgery, University of Cologne, Kerpener Strasse 62, 50937 Cologne, Germany; ruth.lang-roth@uk-koeln.de; 5 Department of Neonatology, Faculty of Medicine, University Hospital Cologne, Kerpener Strasse 62, 50937 Cologne, Germany; bernd.roth1@uk-koeln.de

**Keywords:** newborn hearing screening, quality control, benchmarks, population-based screening, hospital-based screening

## Abstract

Regular reporting of quality control is important in newborn hearing screening, ensuring early diagnosis and intervention. This study reports on a population-based newborn hearing screening program in North-Rhine, Germany and a hospital-based screening at a University Hospital for 2007–2016. The two-staged ‘screening’ and ‘follow-up’ program involving TEOAE and AABR recruited newborns through participating birth facilities. Results were sent to the regional tracking center, and the data were analyzed based on recommended benchmarks. The percentage of newborns from the participating birth facilities in the region increased from 1.4% in 2007 to 57.5% in 2016. The 10-year coverage rate for these newborns was 98.7%, the referral rate after a failed two-step screening was 3.4%, and the lost-to-follow-up rate was 1%. At the hospital, >95% of the screened newborns completed screening within 30 days, the 10-year referral rate was 5%, and 64% were referred within 3 months of age. The median time for screening completion was 6 days after birth, for referral it was 74 days after birth, and for diagnosis it was 55 days after birth. Regional–centralized tracking centers with uniform structure are necessary for proper quality control. Obligatory participation of birthing facilities and quality reports may improve performance, but the recommended quality criteria need considerable financial and infrastructural expenditure.

## 1. Introduction

Permanent hearing loss in newborns and infants is one of the leading congenital disabilities, presenting a significant burden worldwide. The prevalence of unilateral or bilateral hearing loss of 40 dB or more is estimated to be 1.86 in 1000 live births in high-income countries [1,2,3,4]. In Germany, the prevalence of bilateral hearing loss among newborns is estimated to be 0.16%, and unilateral hearing loss is estimated to be 0.07% [5,6].

Early detection and intervention in the form of a treatment or hearing rehabilitation is desirable and should be introduced before the 6th month of age for increased quality of life, to prevent speech impediments, and to help with language skills and communication [7]. Therefore, the Joint Committee on Infant Hearing (JCIH) and the WHO underline the need for early screening of hearing loss and intervention through a universal, mandatory neonatal hearing screening program within the 1st month of age [8,9]. The JCIH in the year 2000 recommended the 1-3-6 plan, which consists of three goals: (1) screening all infants no later than 1 month of age; (2) ensuring diagnostic audiological evaluation no later than 3 months of age for those who do not pass the screening; (3) enrolling infants identified with hearing loss in early intervention services no later than 6 months of age [10]. The 1-3-6 plan was amended to a 1-2-3 plan by the JCIH in 2019 suggesting that the programs meeting the 1-3-6 goal should strive to achieve the 1-2-3 goal [9,10].

The screening programs involve the use of automated transient otoacoustic emissions (TEOAE) and automated auditory brainstem response (AABR) tests, two non-invasive neurophysiological methods to identify newborns who might have hearing loss. These non-invasive tests have led to substantial improvement of the timely detection and initiation of treatment in infants with sensorineural hearing loss within early infancy [11].

Newborns in Germany have been screened for hearing loss already in regional pilot projects since the end of the 20th century, prior to the introduction of the universal hearing screening program in 2009 [12]. Within this framework, databases of screening details and results are handled by 16 regional tracking centers (TCs), spread across the federal states in Germany [13]. For the North-Rhine region, the tracking center is located in Cologne and was established already as a pilot study in 1988 before the initiation of the universal hearing screening program [14]. TCs handle the information pertaining to the screening test results and track the newborns to reduce lost to follow-ups. They connect the regional birth facilities with the regional pediatric audiology departments, which perform the necessary diagnostics and treat newborns who do not pass the hearing screening tests [15].

Quality control is the backbone of such screening programs, ensuring that they function effectively. In the context of hearing screening programs, this means quick and accurate documentation of the patient data, rapid and effective patient tracking mechanisms, and well-functioning follow-up units [16]. The standard of such quality control is measured using the time needed to complete screening, tracking and follow-up processes, and coverage rates. These benchmarks function as crucial indicators in measuring program success, while regular and transparent reporting is essential in maintaining quality. However, studies on program quality control were difficult to identify in the literature; data were outdated and mostly did not reflect the general population [17,18,19,20]. Indeed, there is substantial work to be done in improving the current screening systems, despite hearing screening having been universal for almost a decade in most high-income countries, making the need for quality control studies extremely important.

This study was two-fold. First, we focused on the population-based data from the registry of the universal newborn hearing screening program for the North-Rhine region of Germany involving the participating hospitals for the years from 2007 to 2016. The second part focused on data from a hospital-based screening conducted at a University Children’s Hospital, which also participated in the aforementioned universal screening program with its designated screening and follow-up center (DSFC). The performance of the programs—both hospital-based and population-based—was analyzed for the years from 2007 to 2016 in accordance with the benchmark indicators as suggested by the JCIH in 2000.

## 2. Methods

### 2.1. Recruitment and Structure of Hearing Screening Program

The North-Rhine Universal Newborn Hearing Screening program is a two-staged process consisting of ‘screening’ and ‘follow-up’, using two screening tests, TEOAE and AABR. Recruitment is performed by treating physicians and nurses in the obstetric and pediatric units involved in postnatal healthcare. Written parental informed consent is needed for recruitment. Once recruited, an anonymous personal identification code is automatically created for every newborn, and the data on birth conditions, as well as family history for sensorineural hearing loss are documented in the registry.

Screening is usually completed before discharge of healthy newborns within the first days of life. This bilateral hearing screening includes two phases: S1 and S2. In S1, screening of all healthy newborns, without risk factors, is performed using only TEOAE. Sick newborns or ones with risk factors are directly tested with AABR as a part of S1. Healthy newborns who did not pass TEAOE are tested with AABR either before discharge or before the age of 6 weeks. TEOAE tests and AABR at 35 dB HL are both executed inserting an ear plug consecutively in the ears, one ear at a time, preferably during sleep. All of the tests during S1 take place at obstetric clinics, newborn wards, and regional birth facilities and are facilitated by nurses trained for carrying out the tests.

**Figure 1:**
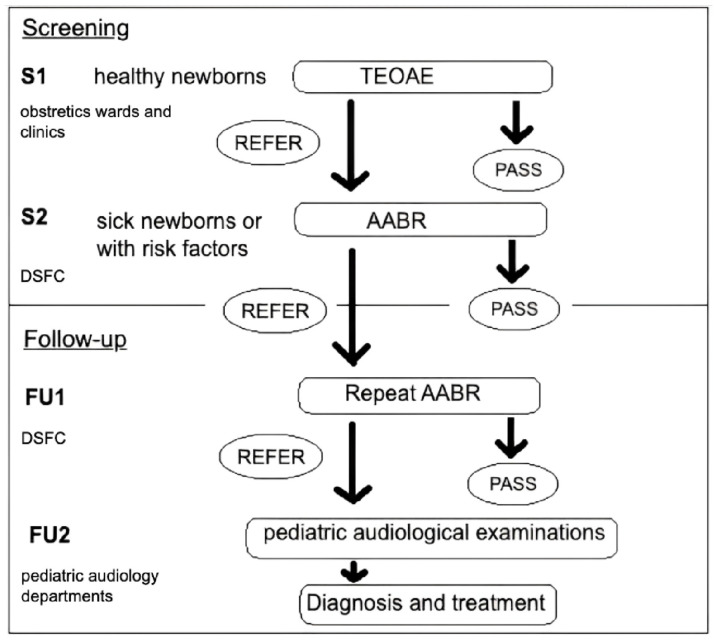
Two-staged newborn hearing screening program in North-Rhine. S1: screening phase 1; S2: screening phase 2; FU1: Follow-up phase 1; FU2: Follow-up phase 2; DSFC: designated screening and follow-up center; TEOAE: automated transient otoacoustic emissions; AABR: automated auditory brainstem response.

The S2 phase starts when a birth facility or an obstetric clinic is either not certified or fails to perform an AABR test during the S1 phase when needed. Then the newborn is sent to a certified designated screening and follow-up center (DSFC) (not to be confused with TC) for a short-term appointment for the AABR test. They primarily do AABR tests (initial test designated as S2 and repeat test designated as FU1). This is because very few regional birth facilities and hospital wards in reality are equipped to perform an AABR test. These DSFCs bridge this gap in infrastructure so that not all newborns who fail TEOAE tests are directly referred to pediatric audiology departments. This reduces the referral rate and in turn a significant burden on the tertiary pediatric audiology departments. The results of S1 (from obstetric clinics and regional birth facilities) and S2 (from DSFC) are sent to the TC where further ‘tracking’ of the newborns with failed screening takes place. The parents and caregivers are then contacted for further ‘follow-up’ appointments (Figure 1).

‘Follow-up’ in turn includes two phases (FU1 and FU2). The first phase (FU1) involves re-testing of the infants who failed ‘screening’ and is performed at the DSFC, using AABR at 35 dB HL [21]. The DSFC notifies the TC about the results of their examination. The follow-up process ends here for infants who failed ‘screening’ but passed FU1. Thus, these centers not only perform the S2 but are also involved in the first phase of the follow-up (FU1) making sure these newborns are not lost in the process especially when repeat tests are needed.

The second phase of follow-up (FU2) involves infants who failed FU1 and shows the referral rate to the hearing screening center [21]. FU2 takes place in specialized pediatric audiology centers, where a protocol of diagnostic tests performed by pediatric audiologists follows to ascertain the etiology of the failed hearing screening. It is important to note that a pediatric audiology specialist examines the newborn only when the newborns are referred to FU2. When a final diagnosis is made, the regional TCs are notified, and the diagnosis is entered into the database.

### 2.2. Coverage Rates and Benchmarks for the Whole of the North-Rhine

Since not all birth facilities in North-Rhine participate and actively recruit newborns in the program, there are difficulties assessing the coverage rate of the universal newborn hearing screening program. In this study, we assessed and reported the coverage rate among the participating facilities and also compared this to the number of live births in North-Rhine throughout the ten-year duration of the study. Parents can refuse either hearing screening itself or only the data transfer of the results from the screening to the tracking center. These both were analyzed together as parental refusal. The rate of lost to follow-up (LTF) infants was also analyzed. This is when the screening process was started, but no further contact could be established for further tests despite the initial test results being questionable.

### 2.3. Benchmarks for the Newborns Screened at the University Hospital and Results from Regional Designated Screening and Follow-Up Center (DSFC)

The second part of the study focuses only on the newborns screened at the University Children’s Hospital as a part of the universal screening program. The university hospital also houses the DSFC and a tertiary pediatric audiology department handling the diagnosis and treatment of ‘referred’ newborns. This DSFC participated in the program throughout the ten-year period, following up on newborns who failed ‘screening’, performing both the S2 and FU1 parts of the program. It is thus intimately involved with every step of the follow-up process of the newborns screened at the University Children’s Hospital, and the benchmarks could be analyzed in detail.

Three benchmarks were analyzed for the newborns screened at the University Children’s Hospital, as introduced by the JCIH in 2007 [10]. According to the American Speech-Language-Hearing Association (ASHA), based on the JCIH goals, the percentage of newborns in a region but also for every unit, who complete ‘screening’ (S1 and S2) by 1 month of age should be 95%, while no more than 4% of newborns should be referred for diagnostic pediatric audiologic evaluation (FU2), which should take place within 3 months of age. These benchmark indicators are directly or indirectly referring to a series of healthcare quality factors, such as the evolution of the coverage rate during the ten years of study, the number of LTF cases, and the average time needed for the completion of ‘screening’ (S1 and S2) and for the completion of FU1 and FU2, as well as for the final diagnosis. These benchmark indicators were calculated as follows:

1. The number of newborns completing ‘screening’ (S1 and S2) by the end of the 1st month of age. The suggested benchmark is ≥95% of the newborns.

(1) $$\mathrm{Newborns}\;\mathrm{screened}\;\mathrm{within}\;1\mathrm{st}\;\mathrm{month}\;(\%)=\frac{\mathrm{Newborns}\;\mathrm{screened}}{\mathrm{Number}\;\mathrm{of}\;\mathrm{births}\;\mathrm{in}\;\mathrm{the}\;\mathrm{population}\;\mathrm{served}}\times 100$$

2. The number of newborns who are referred for FU2 at pediatric audiologic centers (the referral rate). The suggested benchmark is ≤4% of the newborns.

(2) $$\mathrm{Newborns}\;\mathrm{referred}\;\mathrm{to}\;\mathrm{ped}.\;\mathrm{audiology}\;(\%)=\frac{\mathrm{Newborns}\;\mathrm{referred}\;\mathrm{to}\;\mathrm{ped}.\;\mathrm{audiology}}{\mathrm{Number}\;\mathrm{of}\;\mathrm{births}\;\mathrm{in}\;\mathrm{the}\;\mathrm{population}\;\mathrm{served}}\times 100$$

3. The number of newborns who failed the screening test and were referred (referral rate) for a comprehensive diagnostic audiologic evaluation by 3 months of age. The benchmark for this indicator according to ASHA is 90% [10]. This is to see how fast the newborns that failed the screening are referred to pediatric audiology departments. This indicator depicts the quality of cooperation between the designated peripheral center and the pediatric audiology department they are cooperating with. In Germany though, the goal set by the ‘Gemeinsamer Bundesausschuss’ (G-BA) is 100% for all physically healthy newborns who fail the hearing screening [12].

(3) $$\mathrm{Newborns}\;\mathrm{visiting}\;p.\;\mathrm{audiologist}\;\mathrm{within}\;3m\;(\%)=\frac{\mathrm{Newborns}\;\mathrm{visiting}\;p.\;\mathrm{audiologist}\;\mathrm{within}\;3m}{\mathrm{Newborns}\;\mathrm{referred}\;\mathrm{to}\;p.\;\mathrm{audiologist}}\times 100$$

4. A fourth indicator with a benchmark of ≥95% has been suggested by the JCIH, referring to the percentage of infants obtaining hearing amplification within 1 month of confirmation of hearing loss for families choosing that option [10]. In Germany though, this benchmark is extended to up to the 6th month of life [12]. Since this benchmark does not refer to the performance of the designated peripheral center, and rather the screening program as a whole and the pediatric audiology department, it was not analyzed in this study.

### 2.4. Statistical Analysis

A standard inferential chi-square analysis was performed to compare the results throughout the years of the study. A p value of less than 0.05 was considered statistically significant. STATA 14.0 (StataCorp, Texas, USA) was used to perform the analysis for this study.

## 3. Results

### 3.1. Newborn Hearing Screening in the Region of North-Rhine

The average number of live births per year in North-Rhine for years 2007–2016 was 81,000, with a significant increase in the last years. As seen in Table 1, the number of participating hospitals increased over the years, which is reflected in the increase in ‘eligible’ newborns defined as the newborns born at these facilities. The ‘eligible’ newborns increased significantly from 1.4% in 2007 prior to the implementation of universal screening, to 57.4% in the year 2016. From the 373,261 newborns who were born at the participating birth facilities, 368,463 newborns (98.7%) were screened in North-Rhine during the 10-year period. The participation of 1504 newborns (0.4%) in the program was denied by their parents or caregivers, and this rate has remained less than 1% over the years (Table 1). Among the screened newborns, 12,461 newborns (3.4%) were sent for FU2 (referral rate). Another 3656 infants (1.0%) dropped out from the screening program for unreported reasons or could not be tracked anymore and were classified as LTF (Table 1). Among the screening facilities, the coverage rate as well as the LTF rate have been constant. However, the rate of newborns being referred for FU2 (referral rate) was the lowest in 2010 at 2.4% and was found to increase significantly throughout the following years (*p* = 0.021). We still consider the differences in absolute numbers across the years very small and not relevant (Table 1).

### 3.2. Newborn Hearing Screening at a University Children’s Hospital

From 2007 to 2016, a total of 15,327 children were born in the University Hospital where the study was conducted. Another 3637 newborns were transferred within the first two days of life from other hospitals to the University Hospital. From these, a total of 2368 infants (12.5%) failed S1 and were sent to the DSFC and were provided with an appointment for S2 or FU1. However, the parents could decide not to attend the appointment or choose a different center. Of the 2368 infants, 300 (12.7%) infants received S2 screening, and 2068 (87.3%) infants received FU1. The detailed number of infants examined, as well as the total population serviced per year, are presented in the Supplementary Materials.

**Figure 2:**
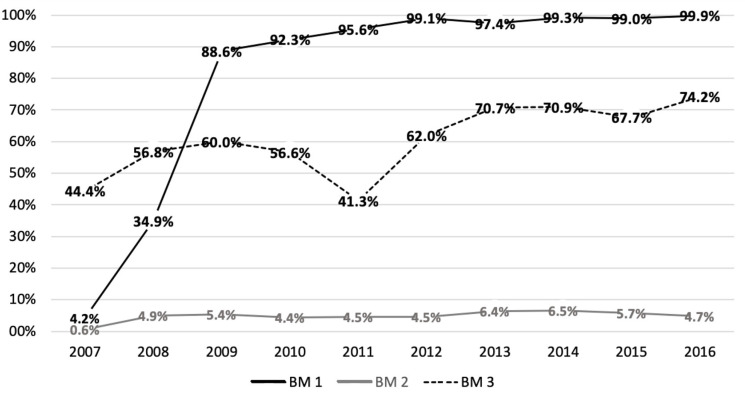
Newborn hearing screening at University Children’s Hospital for the years 2007–2016 with Benchmark indicators (BM) 1, 2, and 3.

The benchmark indicators for the performance of the University Children’s Hospital are presented by year in Figure 2. The number of newborns who completed screening by the end of the 1st month of age (Benchmark 1) increased rapidly from 34.91% in 2008 (the year before establishment of universal hearing screening) to 99.88% in 2016, while the proposed benchmark of 95% was reached in 2011. Regarding the number of newborns who were referred for pediatric audiologic evaluation FU2, that is the referral rate (Benchmark 2 goal <4%), the percentage of referrals showed an increase after implementation of the universal screening program and stayed above 4% in the following years. The third indicator (Benchmark 3), pertaining to the timeline of the referred newborns with the goal of 3 months of age, was found to improve rapidly in the first years of implementation of the universal hearing screening, as well as in the following years, up to 74.2% in 2016, but the center did not reach the JCIH goal. However, we realized that a lot of infants visited the pediatric audiologist just a few days after this deadline. Thus, when we also assessed the number of newborns visiting the audiologist within 4 months of life, it was found to increase significantly up to 85.8% in 2016.

### 3.3. Performance of the Regional DSFC and the Tertiary Pediatric Audiology Center

In total, for the 10-year period of the study, 2368 newborns from the university hospital were sent to the DSFC. The mean time that was required for the completion of ‘screening’ (S1 and S2) for these children was 28.2 ± 51.6 days of life with a median of 6 days. The mean time for the completion of the FU1 was 60.8 ± 78.8 days, and the median was 43 days. The mean and median times for FU2 completion were 104.3 ± 110.8 and 74 days, respectively (Table 2). The mean time needed for the completion of the whole program for the 2368 infants was 80.5 ± 86.5 days (2.6 months), and the median was 55 days (1.8 months).

## 4. Discussion

The universal newborn hearing screening (NHS) was established in Germany by the Federal Joint Committee (‘Gemeinsamer Bundesausschuss’, G-BA) in 2009 [12]. The program is based on the ‘1-3-6 plan’ prescribed by JCIH in the year 2000, which is more suited for population-based screening programs. Germany, being a federal state, although executive decisions about screening protocols and implementation occur at a national level, has regional health authorities at each state ultimately deciding and overseeing implementation [22,23]. Thus, there is a slight difference among the federal states in their organization of the screening, and this difference is also reflected in how benchmarks are calculated [24].

In this context, when we look at the quality criteria prescribed by the Federal Joint Committee in the Children’s Directive (‘Kinder-Richtlinie’), we see that they focus specifically on the screening centers and are hospital-based. These quality criteria are as follows: 1. at least 95% of children born in a hospital should undergo hearing screening (‘coverage’); 2. at least 95% of children who failed the initial screen should receive an AABR rescreening at the same hospital before being discharged; and 3. the proportion of children with findings requiring follow-up after being discharged from the hospital (referral rate) should not exceed 4% [12].

### 4.1. Problems in Reporting Coverage Rate

One of the important differences between hospital-based quality criteria issued by the Children’s Directive in Germany and the JCIH goals is how the coverage rate is reported. Coverage rate is the percentage of newborns screened out of those who are eligible for screening in a particular area. The definition of the eligible population varies, with JCIH describing the number of births in the population as the eligible population, whereas the Children’s Directive lists it as the newborns born in a hospital. This in turn results in different calculations making it difficult to compare or gather the outcomes from various hospitals and hearing screening centers. For example, a study in Bavaria reported a coverage rate of 98.7% of children born in Bavaria for the year 2016, which is population-based [25,26]. Similarly, a population-based study in the federal state of Saxony-Anhalt reported a coverage rate of 98% and above for the years 2011–2015 based on the live births in the state [25]. In Westphalia-Lippe, the newborn hearing screening was established in 2007. A study from that region, reporting on both population- and hospital-based screenings, like ours, shows that a coverage rate of 95% was only achieved from 2010 onwards [27]. Like these studies, the coverage rate for the whole of North-Rhine in this study was also extremely high (98.7%) for the 10-year period. But this does not reflect the real coverage rate based on the number of live births in this region, since it considers the number of births in the participating hospitals only as the eligible population. This is because not all birth facilities/gynecological hospitals participated in the program, which involves co-operation with the regional TC. We see that other regions in Germany face similar problems [20,28]. The number of participating hospitals and birth facilities did show a significant increase in North-Rhine in the first years of the program but reached a plateau around 2014. This does not mean that the newborns in non-participating birth facilities and hospitals are not screened, since the responsibility for carrying out the screening lies with the head of the maternity ward of these hospitals. In case the birth takes place outside the hospital, it is the responsibility of the midwife or doctor accompanying the birth to arrange for hearing screening. When this does not happen, the child’s pediatrician is obliged to check until the fifth pediatric examination (U5), which usually takes place at about the sixth month of life [29]. But these newborns do not enter the databank of the regional TC, and thus we can only speculate about the screenings performed. This shows that there is no clear divide between the federal states where screening is hospital-based and where screening is population-based. In the regions where screening is hospital-based, there are still centralized TCs. But the cooperation of hospitals with the TC being non-mandatory despite the hearing screening being universal presents a common problem in these regions [22].

The individual hospital-based 10-year coverage rate on the other hand was much less (85%), but the yearly rate drastically increased over the years from 4.2% in 2007 to 99.9% in 2016, with a marked increase in the year 2009 when the universal hearing screening program was established. Similarly, a multicentric hospital-based study in Germany from 170 hospitals across four federal states reported an average coverage rate of 91.4% for years 2009-2012 with increased rate of 95.3% in the last year [13].

### 4.2. Benchmarks for Population and Hospital-Based Screenings

For this study, we specifically chose the ASHA-prescribed benchmarks since we believe that these present an amalgamation of JCIH goals and Children’s Directive-prescribed quality criteria [10]. Strictly taken, the JCIH goals are more suited for population-based programs where every child born enters the central databank and is followed up in case of failures in screening. On the other hand, the Children’s Directive-prescribed quality criteria are specific for hospitals and better suited for our setting. The Children’s Directive prescribes that 95% of children who failed the initial screening should receive an AABR rescreening at the same hospital before being discharged. This also proved to be an important problem in our setting, because a lot of birth facilities did not have trained personnel to perform the test [21]. This became a separate step in the process (S2), which was taken over by the so-called DSFC, adding to the burden of the system.

The investigation of the ASHA benchmark indicators gave us important information regarding the performance of the individual DSFC, as well as the interaction with the cooperating pediatric audiology departments and the regional TC. It is obvious that the implementation of the universal screening led to a dramatic increase in all three benchmarks, especially Benchmark 1. In the years following, prioritization of the hearing screening processes within the Department for Pediatrics and the goal not to leave any infant unscreened led to an outstanding increase in the first benchmark indicator. Other studies from Germany and internationally have shown similar results [30,31].

Regarding Benchmark 2, the general referral rate for the North-Rhine region as shown in Table 1 was mostly within the set goal (<4%). But the hospital-based referral rate from the University Children’s Hospital in our study noted a very high referral rate. Referral rates seem to vary considerably based on the type of screening program and the population involved, especially between low- and high-risk newborns. They have been reported to be lower when only AABR was used instead of the two-step TEOAE and AABR protocol [21,32,33]. We believe that the main reason for such referral rates at the hospital was due to the presence of increased high-risk newborns, which is evident from both the transfer of many sick infants and the use of two-step TEOAE and AABR protocol. Regardless, maintaining a lower referral rate is essential for a cost-effective screening. Mackey et al. from EUSCREEN Foundation report that from 47 countries or regions, the referral rate from step 1 was 6–22% where screening may be performed<24 h from birth, 2–15% for >24h, and 4% for >72h. Referral rates to diagnostic assessment averaged 2.1% after one to two steps using TEOAE only, 1.7% after two steps including AABR, and 0.8% after three to four steps including AABR [34]. This shows that referral rates throughout the world across programs are extremely varied and that different reference points are used to calculate them thus making it difficult for any comparison.

When it comes to Benchmark 3, although the average time needed to refer an infant to a pediatric audiologist and organize an appointment showed improvement, the goal of early detection of hearing loss within 3 months of age is far from being reached. This is not dissimilar to other quality reports. Awad et al. reported that 62% of infants were diagnosed with hearing loss by 3 months of age in a large metropolitan children’s hospital in the US [35]. Wang et al. showed that when combined with genetic screening a higher rate of detection of hearing loss could be achieved by 3 months of age among referrals from the initial hearing screening [36]. A global survey using questionnaires from individuals involved in hearing screening from 158 countries reported that the average age at diagnosis of permanent hearing loss was 4.6 months for screened children [18]. Our study also showed that there was an improvement in the number of infants who needed FU2 that were sent within the expected 3rd month (Benchmark 3) to pediatric audiologists. This number increased even more when the time goal was set to the 4th month of age. The improvement of this benchmark was found to coincide with an attempt to improve the documentation of waiting times to perform the screening and to refer the infants to the audiologist. But the 3-month goal seems difficult to achieve even in countries with established screening programs.

### 4.3. Regional DSFC and the Tertiary Pediatric Audiology Center

In hospital-based screening settings, coordinating FU1 and FU2 appointments needs proper cooperation between different departments, especially the DSFC and the pediatric audiology department. The median age in our study was 6 days until completion of screening, 43 days at until first follow-up, 74 days until referral, and 55 days until diagnosis for all the newborns that were processed at the regional DSFC and the tertiary pediatric audiology center. There is unfortunately very little information in the literature regarding these numbers in case of hospital-based screening. The population screening of the NHS newborn hearing screening program in England reported similar results: the median age was 9 days at screen completion, 30 days at entry into follow-up, 49 days at confirmation, and 50 days at referral to early intervention [31]. For further comparison, a tertiary pediatric audiology center from Germany reported that newborns with abnormal initial screening or risk factors were presented for referral at around 5.3 weeks and at 8 weeks of median age, respectively, while newborns that reported directly to them for initial screening were presented at a median age of 4.6 weeks. Permanently or transiently hearing-impaired patients were diagnosed at a median age of 11.4 and 23.1 weeks and those with normal hearing at 5.9 weeks [28]. This again shows considerable differences in the organization of universal hearing screening programs and the need for more quality control studies.

### 4.4. Limitations and Strengths

Not all departments of the University Children’s Hospital participated from the start in the hearing screening processes. For example, initially only newborns admitted in the neonatal intensive care unit were screened. Generalizability is a major drawback since the Children’s Hospital is a tertiary care center with a relatively big intensive care unit, and thus a big part of the population involves premature and sick infants. This not only complicates the process of hearing screening but also increases the referral rate. On the other hand, some infants were transferred to other hospitals before the hearing screening processes were completed. One should also critically point out that, for example, in 2016 only 39 out of 69 birth centers from the region took part in the centralized screening program, and therefore only an incomplete statement on benchmarking is possible. The inconsistent policy-making in any federalized political system must be mentioned here, and further improvement of such health systems needs to be addressed in the future.

## 5. Conclusions

Despite establishment of a universal newborn hearing screening program, continued education of parents and health workers about screening and thus early detection of hearing loss is needed. Establishment of regional–centralized hearing screening and tracking centers with a uniform structure is necessary for effective quality control.

The implementation of obligatory benchmarking for all follow-up centers on a national level is a measure that would significantly improve the performance of universal newborn hearing screening. Nevertheless, both the 1-3-6 and the newly amended 1-2-3 quality criteria are very demanding and can only be achieved with considerable financial and infrastructural expenditure.

## Figures and Tables

**Table 1:** Newborn hearing screening in North-Rhine (NR), recruited and screened and in the North-Rhine Hearing Screening Centre for the years 2007–2016. DSFC: Regional designated screening and follow-up center, CR: Coverage rate at the participating birth facilities, PR: Parental refusal, RR: Referral rate, LTF: Lost to follow-up.

	2007	2008	2009	2010	2011	2012	2013	2014	2015	2016
Live Births NR	80,828	80,840	78,149	80,107	77,987	79,566	79,732	84,736	87,499	94,749
Birthing centers *	6	36	56	61	60	64	62	69	68	69
DSFC *	5	7	16	23	29	30	33	38	37	39
Newborns eligible ^+^	1.4%	14.3%	41.3%	46.7%	50.1%	51.9%	53.8%	54.3%	57.5%	57.4%
CR% ^#^	98.0%	96.3%	98.2%	98.3%	98.6%	98.8%	98.9%	98.9%	98.8%	99.2%
PR%	0.8%	0.6%	0.03%	0.3%	0.4%	0.3%	0.3%	0.4%	0.6%	0.4%
RR%	5.2%	2.9%	4.1%	2.4%	2.9%	3.3%	3.1%	3.3%	3.4%	3.9%
LFT%	NA	1.8%	2.0%	1.6%	1.1%	0.8%	1.0%	0.8%	0.7%	0.5%

* Birthing centers and DSFCs volunteering to be part of the regional tracking and screening center and sending the results of the screening to the tracking center. + percentage of newborns born at the participating birthing centers and thus eligible for screening. # percentage of newborns screened among the newborns eligible. NA: Data not available.

**Table 2:** Time taken for the hearing screening test and follow-up and for diagnosis among newborns from the designated screening and follow-up center and the pediatric audiology center for the years from 2007 to 2016 (N = 2368); SD: Standard deviation.

	Mean Days of Life (d)± SD Median (d)
Days since birth for completion of screening (S1 + S2)	28.2 ± 51.6 6
Days since birth for completion of FU1	60.8 ± 78.8 43
Days since birth for completion of FU2	104.3 ± 110.8 74
Time interval between FU1 and FU2	40.2 ± 83.8 19
Days of birth for diagnosis	80.5 ± 86.5 55

## Data Availability

The data that support the findings of this study are available from the authors, but restrictions apply to the availability of these data, which were used under the license of the University Hospital of Cologne and the medical ethics committee for the current study, and so are not publicly available for reasons of data protection and sensitivity. Data are, however, available from the authors upon reasonable request and with permission from the University Hospital of Cologne and the medical ethics committee.

## Supplementary Materials

The following supporting information can be downloaded at: https://www.mdpi.com/article/10.3390/ijns9040061/s1, Table S1. Newborn hearing screening in North-Rhine, screened and recruited in the North-Rhine Hearing Screening Centre for the years 2007–2016, Table S2. Newborn hearing screening at University Children’s hospital for the years 2007–2016 with Benchmark indicators 1, 2, 3 an assessment within 4 months).
