# Supplementary material for: Universal Newborn Hearing Screening Program: 10-Year Outcome and Follow-Up from a Screening Center in Germany
Source: Int J Neonatal Screen. 2023 Oct 23;9(4):61. doi: 10.3390/ijns9040061 (PMC10594500; doi:10.3390/ijns9040061)
Supplement: Supplementary file 1 [file IJNS-09-00061-s001.zip › IJNS-2612347-supplementary.pdf]

Supplementary material

Table S1. Newborn hearing screening in North-Rhine, screened and recruited in the North-Rhine Hearing Screening Centre for the years 2007 – 2016. LTF: lost to follow-up, FU: follow-up, CR: coverage rate.

Year	**Live births in region**	**Birthing/ DSFC centers participating**	**Newborns eligible for screening* (%)**	**Newborns participated (CR in %)**	**Parental refusal (%)**	**FU2/Referral rate (%)**	**LTF (%)**
Total	NA	NA	NA	368463 (98.7%)	1504 (0.4%)	12461 (3.4%)	3656 (1.0%)
2007	80828	6 /5	1153 (1.4%)	1131 (98%)	9 (0.8%)	59 (5.2%)	Data not available
2008	80840	36 / 7	11593 (14.3%)	11165 (96.3%)	73 (0.6%)	325 (2.9%)	212 (1.8%)
2009	78149	56 /16	32252 (41.3%)	31659 (98.2%)	10 (0.03%)	1293 (4.1%)	658 (2.0%)
2010	80107	61 /23	37437 (46.7%)	36798 (98.3%)	109 (0.3%)	873 (2.4%)	608 (1.6%)
2011	77987	60 /29	39087 (50.1%)	38540 (98.6%)	149 (0.4%)	1126 (2.9%)	444 (1.1%)
2012	79566	64 / 30	41307 (51.9%)	40796 (98.8%)	154 (0.3%)	1363 (3.3%)	311 (0.8%)
2013	79732	62 / 33	42912 (53.8%)	42441 (98.9%)	150 (0.3%)	1332 (3.1%)	422 (1.0%)
2014	84736	69 / 38	46060 (54.3%)	45585 (98.9%)	227 (0.4%)	1510 (3.3%)	364 (0.8%)
2015	87499	68 /37	50327 (57.5%)	49741 (98.8%)	308 (0.6%)	1688 (3.4%)	368 (0.7%)
2016	94749	69 /39	54379 (57.4%)	53969 (99.2%)	230 (0.4%)	2110 (3.9%)	261 (0.5%)

* Newborns born at the participating birth centers and gynecological clinics

Table S2. Newborn hearing screening at University Children's hospital for the years 2007-2016 with Benchmark indicators 1, 2, 3 an assessment within 4 months), NA: not applicable

	2007	2008	2009	2010	2011	2012	2013	2014	2015	2016	Total
**Total population screened**	1436	1501	1487	1574	1765	2057	2081	2323	2194	2546	18964
(Births in the university hospital + Transfers)	1152+ 284	1244+ 257	1212+ 275	1337+ 237	1435+ 330	1494+ 563	1693+ 388	1845+ 478	1809+ 385	2106+ 440	15327+ 3637
**Newborns referred to S2 or FU1 at DSFC**	59	256	289	253	226	209	251	268	298	259	2368
No. of newborns receiving S2/FU1	6/53	141/115	33/265	26/227	40/186	23/186	11/240	7/261	4/294	9/250	300/2068
**Benchmark indicator 1* (%)**	4.2	34.9	88.6	92.3	95.6	99.1	97.4	99.3	99.0	99.9	NA
Newborns sent for FU2 which is Referral rate. **Benchmark indicator 2 (%)**	9<br>0.6	74<br>4.9	80<br>5.4	76<br>4.4	80<br>4.5	92<br>4.5	133<br>6.4	151<br>6.5	124<br>5.7	120<br>4.7	939<br>5.0
Newborns who received F2 within 3 months. **Benchmark indicator 3 (%)**	4<br>44.4	42<br>56.8	48<br>60.0	43<br>56.6	33<br>41.3	57<br>62.0	94<br>70.7	107<br>70.9	84<br>67.7	89<br>74.2	601<br>64.0
Newborns who received F2 within 4 months. (%)	4<br>44.4	44<br>59.5	56<br>70.0	55<br>72.4	47<br>58.6	66<br>71.7	104<br>78.2	117<br>77.4	104<br>83.8	103<br>85.8	700

*Benchmark 1: newborns completing 'screening' (S1 and S2) by 30 days
